# Teppanyaki/Hibachi Pneumonitis: An Exotic Cause of Exogenous Lipoid Pneumonia

**DOI:** 10.1155/2016/1035601

**Published:** 2016-11-14

**Authors:** Franck Rahaghi, Ali Varasteh, Roya Memarpour, Basheer Tashtoush

**Affiliations:** Department of Pulmonary and Critical Care Medicine, Cleveland Clinic Florida, 2950 Cleveland Clinic Blvd., Weston, FL 33331, USA

## Abstract

Exogenous lipoid pneumonia (ELP) is a rare type of inflammatory lung disease caused by aspiration and/or inhalation of fatty substances and characterized by a chronic foreign body-type reaction to intra-alveolar lipid deposits. The usual clinical presentation occurs with insidious onset of nonspecific respiratory symptoms and radiographic findings that can mimic other pulmonary diseases. Diagnosis of ELP is often missed or delayed as it requires a high index of suspicion and familiarity with the constellation of appropriate history and radiologic and pathologic features. We herein report a case of occupational exposure to tabletop “Teppanyaki” entertainment cooking as a cause of ELP, confirmed by surgical lung biopsies in a 63-year-old Asian woman who worked as a Hibachi-Teppanyaki chef for 25 years.

## 1. Introduction

Lipoid pneumonia was first described by Laughlen in 1925, in one adult, one infant, and two children after repeated inhalation of nasopharyngeal oil droplets [1]. Since then, it has been widely recognized and reported in association with many other medications, substances, diseases, and exposures. Although data on the precise incidence of lipoid pneumonia are lacking, autopsy studies have reported an incidence of 1.0–2.5% [2]. Lipoid pneumonias can be classified based on the source of lipids into exogenous and endogenous types. The exogenous type is more common and associated with aspiration or inhalation of fatty substances, whereas the endogenous type usually occurs as secondary bronchial obstruction caused by tumors, bacterial infections, bronchiolitis obliterans, and lipid storage diseases [3–5].

Diagnosis of ELP is based on a strong history of exposure to lipids with a risk for aspiration and/or inhalation, characteristic radiologic features on chest computed tomography (CT) scan, and the presence of intra-alveolar lipids and lipid-laden macrophages on histopathology specimens. While treatment protocols for ELP are poorly defined, a high index of suspicion and early diagnosis can halt the progression of the disease by avoiding further exposure and allowing appropriate and timely supportive care.

## 2. Case Presentation

A 63-year-old Japanese woman was referred to the pulmonary clinic for evaluation of her interstitial lung disease which was initially diagnosed as idiopathic pulmonary fibrosis (IPF). She had no history of smoking, chronic lung disease, or respiratory infections. She complained of a dry cough for two months, mostly at nighttime, and occasionally woke her from sleep. Associated symptoms included mild hoarseness of voice in the early morning, dyspepsia, and mild symptoms of gastroesophageal reflux disease (GERD). She had no associated symptoms of myalgia, arthralgia, or rash and denied any history of dyspnea, chest pain, fever, chills, weight loss, or night sweats. Her past medical history was significant for type 1 diabetes mellitus diagnosed in childhood, dyslipidemia, and cholelithiasis. Her medications included subcutaneous short acting premeal insulin and daily long acting insulin. She was born and raised in Tokyo and immigrated to the United States, where she has lived for the last 30 years, with no history of sick contacts or recent travel and no family history of chronic diseases. She works as manager of a Japanese cuisine restaurant, specialized in tabletop “Teppanyaki/Hibachi” cooking where she also worked as a chef for the last 25 years.

**Figure 1:**
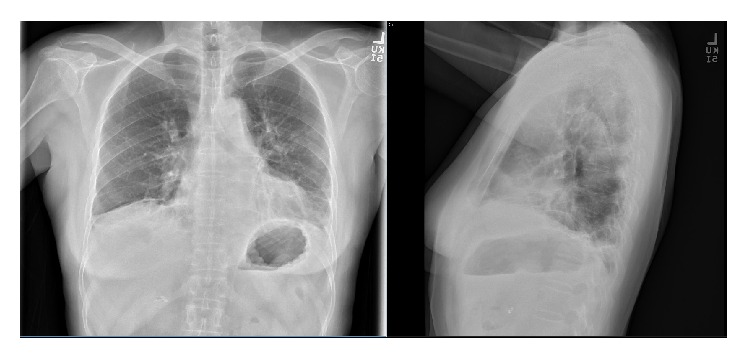
CXR, PA, and lateral view, showing bilateral perihilar and left lower lobe reticular opacities with volume loss.

**Figure 2:**
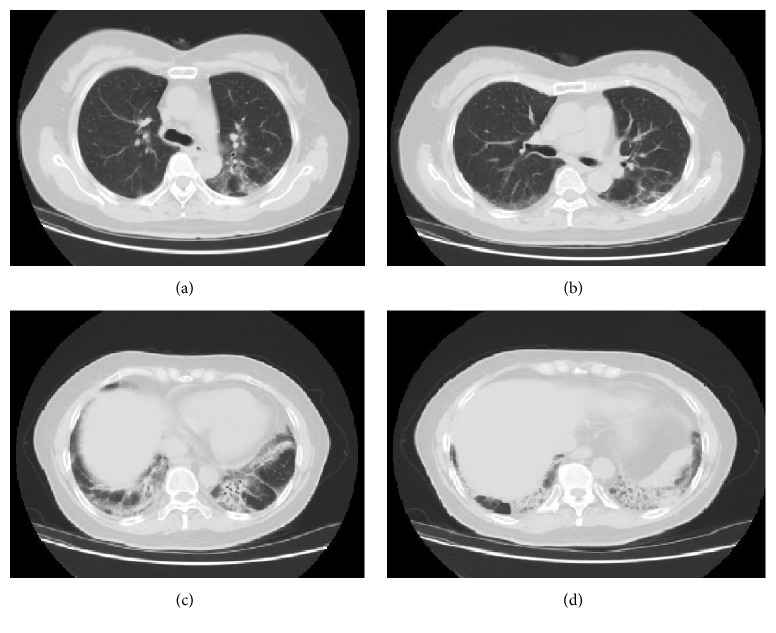
CT chest, axial views, showing bilateral peribronchial, lower lobe predominant ground glass opacities with evidence of early fibrosis as demonstrated by lower lobe volume loss and traction bronchiectasis.

On physical examination, there was no evidence of respiratory distress, and blood pressure was 120/74 mmHg, pulse rate 70, respiratory rate 16, and Oxygen saturation 96% on room air. Breath sounds were equal bilaterally with fine crackles over both lung bases and no wheezing. Remainder of her physical examination was normal. Chest X-ray (Figure 1) showed bilateral patchy middle and lower lung zone patchy and reticular opacities. Pulmonary function test revealed significant restriction: forced vital capacity (FVC): 1.17 liters, 36% of predicted, forced expiratory volume in 1st second (FEV1): 0.98 liters, 40% of predicted, (FEV1/FVC) 84, total lung capacity (TLC): 58% of predicted, and diffusion lung capacity for carbon monoxide (DLCO) being significantly reduced, at 53% of predicted. Chest CT scan showed bilateral peribronchial, lower lobe predominant ground glass opacities with evidence of pulmonary fibrosis (Figure 2).

Differential diagnosis included chronic aspiration related interstitial lung disease (ILD), nonspecific interstitial pneumonia (NSIP), idiopathic or secondary to an underlying connective tissue disease, chronic eosinophilic pneumonia, and IPF. Given the peribronchial distribution of the inflammatory changes on CT scan, hypersensitivity pneumonitis (HP) and sarcoidosis were also considered in the differential as well as other types of granulomatous lung disease such as granulomatosis with polyangiitis (GPA).

Laboratory work-up for autoimmune and connective tissue diseases was negative. Erythrocyte sedimentation rate (ESR), C-reactive protein (CRP), and hypersensitivity pneumonitis panel were all within normal limits. Bronchoscopy was performed with a bronchoalveolar lavage (BAL) and transbronchial lung biopsies. There was no visible airway pathology, and BAL fluid had a total white blood cell (WBC) count of 121 cells/mm^3^, with neutrophil predominance (94%), a low lymphocyte count (2%), and elevated eosinophil count (4%). BAL fluid was negative for AFB and bacterial or fungal pathogens and no malignant cells were identified. Transbronchial lung biopsy samples were deemed nondiagnostic.

**Figure 3:**
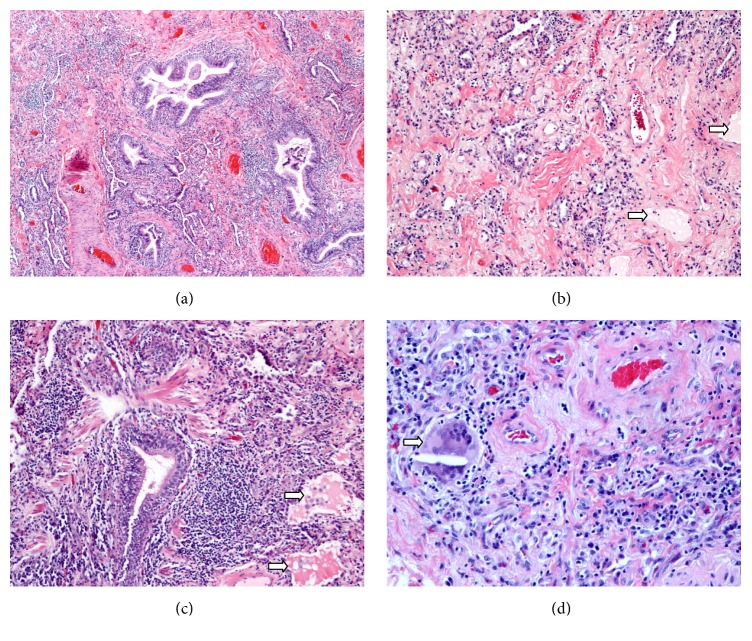
Photomicrographs of lung biopsy with hematoxylin-eosin stain. (a) 100x, chronic interstitial inflammation consisting of dense bronchocentric lymphoplasmacytic infiltrates, with significant interstitial fibrosis. (b, c) 200x, intra-alveolar lipids and lipid-laden macrophages (*arrows*). (d) 400x, a multinucleated giant cell with a cholesterol cleft (*arrow*); an early manifestation in the development of cholesterol granulomas.

A video assisted surgical lung biopsy was performed from the right lower lobe (Figure 3) and showed chronic interstitial inflammation consisting of dense bronchocentric lymphoplasmacytic infiltrates with multinucleated giant cells containing cholesterol clefts and intra-alveolar lipid-laden macrophages. In multiple areas, the infiltrates formed well-circumscribed aggregates around airways, with germinal center-like structures and significant interstitial fibrosis. Based on the above constellation of clinical history and radiologic and pathologic features, a diagnosis of ELP was made and attributed to her heavy and repeated exposure to the inhalation of aerosolized lipids from the tabletop flames, as this was part of her everyday cooking performance for the past 25 years.

The patient was instructed to avoid further exposure to the Teppanyaki/Hibachi grill fumes and flames. She received prednisone 20 mg daily for two months and a proton pump inhibitor twice daily with GERD preventive measures. Repeated imaging eight months later showed mild improvement in the ground glass opacities on CT scan, and repeated pulmonary function testing had a persistent restrictive pattern, yet with marked improvement in DLCO by 12% (from 53 to 65% of predicted).

## 3. Discussion

Exogenous lipoid pneumonia (ELP) is a rare form of pneumonia caused by inhalation or aspiration of fatty substances. It is characterized by a chronic foreign body-type reaction to inhaled exogenous lipid droplets on histologic specimens. ELP has been reported with aspiration of a large variety of lipid containing substances, such as petroleum jelly, mineral oil laxatives, oil based nasal drops, milk, poppy seed oil, and egg yolk. It has also been described in patients with prolonged facial application of petrolatum for erythrodermic psoriasis and even with the excessive use of lip balm and flavored lip-gloss [3, 4].

As lipids float on the surface of gastric fluids, ingested oil based medications or food may enter the airway due to regurgitation and aspiration of gastric contents. Therefore factors that may increase the risk of ELP include extremes of age, structural abnormalities of the pharynx and esophagus (e.g., Zenker's diverticulum, hiatal hernia, and achalasia), psychiatric disorders, recurrent loss of consciousness, and neuromuscular disorders that can cause swallowing dysfunction and/or affect the cough reflex [6].

Occupational exposures that may lead to ELP through inhalation injury include exposures to paraffin, such as paraffin droplets released by machines in cardboard crockery factories, the use of spray paint, plastic production factories and cleaning of new cars protected by paraffin.

Fire eaters' pneumonitis and Diesel siphoner's lung are two recently described occupational exposures that have been considered among the unusual causes for ELP. In fire eaters' pneumonitis, pyrofluids are used by “fire eaters” (performers who spit fire). Kerdan is a petroleum derivative and the most common pyrofluid used by performers. It is characterized by its reduced viscosity and ability to rapidly diffuse throughout the bronchial tree. The mechanism of injury is thought to occur after flame blowing, when the fire-eater takes a deep inspiration, and Kerdan remaining in the mouth can be aspirated [7–10]. Diesel siphoner's lung is a hydrocarbon pneumonitis described in individuals who transfer or steal gasoline from vehicles using a tube or a hose, where the individual draws the fluid with negative inspiratory force, often aspirating small quantities into the airway and lungs, “perhaps exacerbated by the rush of the moment!”, triggering a similar inflammatory pulmonary reaction caused by petroleum hydrocarbons [11–14].

Almost 50% of patients with ELP are asymptomatic. In many cases the disease is discovered by chance, during routine chest imaging. Symptoms are also nonspecific (chest pain, dyspnea, cough, or fever) and vary according to the duration of exposure and the amount and quality of oil aspirated [15].

The characteristic imaging findings of ELP on high resolution CT chest (HRCT) can be summarized into three major categories: (1) alveolar filling (ground glass pattern) with or without crazy paving, often with subpleural sparing, (2) consolidative pulmonary lesion with spontaneous angiogram sign on unenhanced HRCT (pulmonary vessels may spontaneously be visible within the areas of parenchymal filling without IV contrast), and (3) low-density pulmonary consolidation (−30 to −150 Hounsfield units) in a bronchocentric distribution [16–18].

On histopathology, ELP is confirmed by the presence of giant cell granulomas, a bronchocentric chronic alveolar and interstitial inflammation with cholesterol clefts (vacuoles) in alveolar macrophages, similar to the pathologic findings seen in our patient.

BAL may be useful in the evaluation of patients with suspected ELP, where lipid-laden macrophages on cytological evaluation are consistent with the diagnosis. However, false-negative results may occur, and the finding is nonspecific [19]. Slight increase in eosinophil numbers may increase the specificity of BAL in diagnosing ELP [14, 20] which was also seen in our patient, despite the absence of other characteristic BAL findings.

The diagnosis of ELP is based on three important elements, (1) history of exposure to oil, with the risk for aspiration and/or inhalation, (2) characteristic radiologic findings, and (3) the presence of lipid-laden macrophages in sputum, BAL analysis, or histopathology specimens.

In the USA, restaurants serving Teppanyaki cuisine are often incorrectly known as “Hibachi grills.” Modern Teppanyaki grills are typically propane-heated flat surface grills that are widely used to cook food in front of diners. These are commonly confused with the Hibachi barbecue grill, which has a charcoal or gas flame and is made with an open grate design.

Chains of Teppanyaki steakhouses continue to place an emphasis on the chef performing a show for the diners. The chef might juggle utensils, toss an egg up in the air and split it with a spatula, or arrange onion rings into fire-shooting volcanoes. To create a flame or volcano, a grain alcohol similar to vodka is often used. The alcohol is added on top of the oil to light up the volcano or flame and the burning oil sustains it.

We suspect that repeated daily exposure, with close proximity to the oil sustained flames on a tabletop cook surface, can predispose the chef and staff to a lipid inhalation injury causing ELP, as it has been reported with other similar occupational exposures. The problem is likely intensified in the absence of adequate ventilation. Although it is an unusual cause of chronic lung disease, it can produce a severe inflammatory pneumonitis that can progress to irreversible pulmonary fibrosis, as seen in our patient.

First step in the treatment of ELP is to prevent ongoing exposure and provide supportive care. Treatment of underlying risk factor for gastric content or food aspiration is imperative, especially when the mechanism of injury is not of a direct inhalational type. Systemic steroids have been used to slow the inflammatory response but are only supported by anecdotal case reports [21, 22]. Steroids can perhaps be withheld unless the lung injury is severe and ongoing, as demonstrated by the biopsy specimens and/or the extent of ground glass opacities on chest CT. Whole or affected segment lung lavage with an emulsifying liquid has also been used with favorable outcomes in severe cases [23–27].

## 4. Conclusion

Teppanyaki/Hibachi pneumonitis is a type of exogenous lipoid pneumonia that can be triggered by the inhalation of aerosolized lipids when igniting mixtures of alcohol and vegetable oil to create spectacular flames and volcanoes in restaurants with tabletop cooking performances. This injury is perhaps more likely to occur after decades of close and repetitive exposure and may lead to a progressive interstitial lung disease with pulmonary fibrosis.
